# Parent Experiences With Electronic Medication Monitoring in Pediatric Asthma Management: Qualitative Study

**DOI:** 10.2196/25811

**Published:** 2021-04-23

**Authors:** Kristin Kan, Sara Shaunfield, Madeleine Kanaley, Avneet Chadha, Kathy Boon, Carolyn C Foster, Luis Morales, Patricia Labellarte, Deneen Vojta, Ruchi S Gupta

**Affiliations:** 1 Ann & Robert H. Lurie Children’s Hospital of Chicago Chicago, IL United States; 2 Division of Advanced General Pediatrics and Primary Care Department of Pediatrics Northwestern University Feinberg School of Medicine Chicago, IL United States; 3 Department of Medical Social Sciences Northwestern University Feinberg School of Medicine Chicago, IL United States; 4 Institute of Public Health and Medicine Northwestern University Feinberg School of Medicine Chicago, IL United States; 5 UnitedHealth Group Minnetonka, MN United States

**Keywords:** pediatric asthma, digital health, outpatient care, asthma management, pediatric, asthma, parents, caregivers, Bluetooth sensors, inhaler

## Abstract

**Background:**

Electronic medication monitoring (EMM) is a digital tool that can be used for tracking daily medication use. Previous studies of EMM in asthma management have been conducted in adults or have examined pediatric interventions that use EMM for less than 1 year. To understand how to improve EMM-enhanced interventions, it is necessary to explore the experiences of parents of children with asthma, recruited from outpatient practices, who completed a 12-month intervention trial.

**Objective:**

The objective of our study was to use qualitative inquiry to answer the following questions: (1) how did using an EMM-enhanced intervention change parents'/caregivers’ experiences of managing their child’s asthma, and (2) what do parents recommend for improving the intervention in the future?

**Methods:**

Parents were recruited from the intervention arm of a multicomponent health intervention enhanced by Bluetooth-enabled sensors placed on inhaler medications. Semistructured interviews were conducted with 20 parents of children aged 4-12 years with asthma. Interviews were audio-recorded, transcribed, and inductively analyzed using a constant comparative approach.

**Results:**

Interview participants reflected an even mix of publicly and privately insured children and a diverse racial-ethnic demographic. Parents discussed 6 key themes related to their experience with the EMM-enhanced intervention for the management of their child's asthma: (1) compatibility with the family's lifestyle, (2) impact on asthma management, (3) impact on the child’s health, (4) emotional impact of the intervention, (5) child’s engagement in asthma management with the intervention, and (6) recommendations for future intervention design. Overall, parents reported that the 12-month EMM intervention was compatible with their daily lives, positively influenced their preventive and acute asthma management, and promoted their child's engagement in their own asthma management. While parents found the intervention acceptable and generally favorable, some parents identified compatibility issues for families with multiple caregivers and frustration when the technology malfunctioned.

**Conclusions:**

Parents generally viewed the intervention as a positive influence on the management of their child's asthma. However, our study also highlighted technology challenges related to having multiple caregivers, which will need to be addressed in future iterations for families. Attention must be paid to the needs of parents from low socioeconomic households, who may have more limited access to reliable internet or depend on other relatives for childcare. Understanding these family factors will help refine how a digital tool can be adopted into daily disease management of pediatric asthma.

## Introduction

An estimated 6.2 million children in the United States currently have asthma, with 60.3% of them experiencing persistent disease severity [1]. Asthma that is persistent and poorly controlled places children at risk for frequent symptoms of respiratory distress leading to acute unscheduled health care, activity limitations, and school absenteeism [2]. Per national asthma guidelines, children with persistent asthma should be using daily preventive anti-inflammatory medications for symptom control [3,4]. Nevertheless, estimated adherence among US children with asthma to long-term control medications, such as inhaled corticosteroids (ICSs), is 40% or lower [5-9].

New technologies, such as electronic medication monitoring (EMM), allow patients and health providers to digitally track adherence to daily preventive asthma medications. EMM includes a wide range of digital devices, such as pillbox sensors that measure the opening time of medications [10] or inhaler sensors that detect the delivery of an actuation (ie, puff of medication). EMM as a digital tool, accompanied by other patient-centered supports, can also enhance provider-patient communication around chronic disease management. In asthma, studies evaluating EMM have previously focused on the experiences of EMM among adults [11]. Studies of children and adolescents with asthma have been limited to a short duration of EMM exposure (eg, 1 to 6 months) [12-14].

Enhancing pediatric asthma management with digital tools requires understanding parents’ acceptance of the technology over a longer period of use and in clinical scenarios that closely reflect how patients and health providers use EMM. We present findings that explored the use of EMM by parents in a 12-month intervention trial embedded in outpatient pediatric practices. The trial studied the effects of EMM via Bluetooth-enabled inhaler sensors, accompanied by a mobile app in pediatric asthma management [15]. Sensors tracked daily inhaler medication usage, which parents and clinicians could monitor. Our qualitative study explored 2 key questions to ascertain parent experiences of participating in the intervention with EMM: (1) how did using the intervention change parents'/caregivers’ experiences of managing their child’s asthma, and (2) what do parents recommend for improving the intervention in the future?

## Methods

### Sample and Data Collection

We recruited parents from the intervention arm of the Improving Technology-Assisted Recording of Asthma Control in Children (iTRACC) trial for interviews [15]. In the original trial, caregiver and child dyads were eligible if the following criteria were met: (1) child was aged 4 to 17 years; (2) child had experienced at least one asthma exacerbation requiring oral corticosteroids in the year prior to enrollment; and (3) parent reported active prescription of an ICS or combination ICS–long-acting beta-agonist (ICS-LABA) for at least 1 year prior to enrollment. The exclusion criteria were as follows: (1) dyad was non–English speaking; (2) child had a comorbid condition that could interfere with asthma symptom assessment (eg, cystic fibrosis); or (3) dyad was participating in another sensor-based intervention that would interfere with the use of the trial devices.

We used purposive sampling of parents of children aged 4-12 years in the intervention group because only the intervention arm dyads had the smartphone app, sensors, and EMM at their clinics [16]. We did not recruit adolescents for this qualitative study because we anticipated that they would experience a different relationship in asthma co-management with their parents than would younger children. Aligned with purposeful sampling strategies, we aimed for a balanced representation from all 5 clinic sites; public versus private insurance; and 3 general categories of adherence (low, medium, and high), measured by the sensors [16]. Adherence was categorized as low (<30%), medium (30%-70%), or high (>70%) based on the mean daily adherence of the patient to their preventive inhaler medication over a 9-month period. Since the intervention was intended to improve adherence to preventive medications, we wanted to ensure that dyads with low and medium adherence were represented. The qualitative interviews were a separate study from the original trial. Fifty-eight parents from the original trial were found to be eligible for the qualitative study, based on the aforementioned criteria, and 31 agreed to be contacted for further research at trial completion. One parent—the parent of record for the original trial—was contacted for each child. Parents were called and emailed about the qualitative study, and 20 parents were scheduled for an in-person or telephone interview, based on their preference [17]. On average, parents were interviewed 5 months following completion of the trial, and 6 parents indicated a preference for a telephone interview. The study was approved by the hospital's institutional review board (IRB 2016-698), and written informed consent was obtained from all participants. The interview study was funded by the Agency for Healthcare Research and Quality.

### Intervention Description

**Figure 1:**
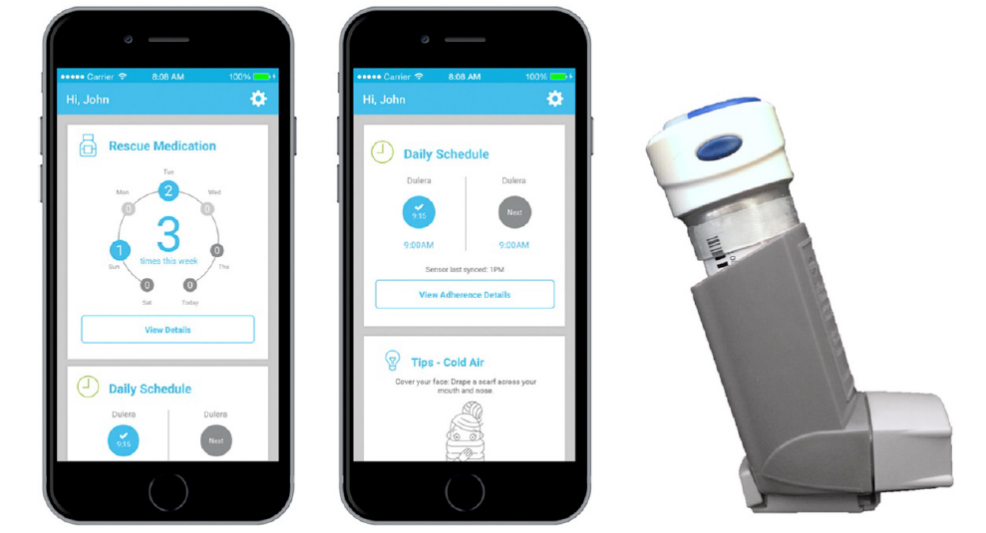
Inhaler sensor and mobile app (Propeller Health).

The iTRACC trial involved a multicomponent health intervention that included (1) Bluetooth-enabled sensors placed on inhaler medications that paired with the parent’s smartphone via a mobile app (Propeller Health), and (2) monitoring through a web portal and follow-up phone calls by clinic staff [15,18] (Figure 1). The EMM technology tracked the use of most ICSs, short-acting beta-agonists (SABAs), and combination ICS-LABAs that were available on the US market. Medication doses could be automatically or manually synced to a smartphone app for parents. Parents set up timed reminders for administering daily ICS medications and were notified by push notifications from the app when medications were missed. They were also provided local daily reports on environmental allergens and summaries of medication adherence upon opening the app. Alerts by email and through a web portal notified health providers if their patients had increased SABA use (ie, >4 uses in a 24-hour period) or decreased ICS or ICS-LABA use (ie, no detected doses in 4 days). Upon receiving the alerts, clinic staff (ie, physician, nurse, or medical assistant) called parents to triage how to improve adherence or discern the cause of increased SABA use. The 12-month randomized clinical trial was conducted from 2016 to 2018 in Chicago, Illinois, and included 5 outpatient practices that served pediatric patients (ie, 2 academic primary care clinics, 1 community primary care clinic, 1 academic pulmonary clinic, and 1 private family allergy clinic). The trial was registered at ClinicalTrials.gov (NCT02994238).

### Interviews

Interviews were conducted between March and July 2019 by trained facilitators (KK, MK, AC, SS, and PL). Participants were compensated US $100 for their time. We conducted 1-hour interviews with parents to explore their experiences with the EMM-based iTRACC intervention using a semistructured interview guide (Multimedia Appendix 1). The guide was designed to explore (1) the intervention’s compatibility with the family’s lifestyle, (2) perceived intervention utility, (3) the intervention’s impact on the child’s asthma management and health, and (4) suggestions for improving the intervention to better meet parents’ needs. Interviews were audio-recorded, transcribed, and deidentified for analysis.

### Data Analysis

Interview transcripts were inductively analyzed via a team-based approach to coding with constant comparison across cases [19-21]. In the first cycle of coding, 4 authors (SS, KK, PL, and MK) with expertise in qualitative research, pediatric medicine, and experience with the iTRACC trial independently conducted descriptive line-by-line coding of one transcript and discussed observations, which informed the development of a preliminary codebook [21,22]. The coders then reviewed a second transcript using the draft codebook and revised the codebook and definitions through group discussion; this same process was conducted on a third transcript. Next, the data set (including transcripts from codebook development) was divided equally among the analysts and independently coded in Dedoose, a cross-platform app for qualitative analysis [23], using the codebook. The codebook was refined throughout the analysis process through team discussion. After finalizing the codebook and coding all transcripts, we conducted second cycle coding using thematic analysis [21,22,24,25]. In this cycle, the text for each code was extracted and reviewed in a “coding review process,” during which the data for each code were reviewed and summarized, and any errors in coding were discussed by the team and corrected. Next, code summaries were reviewed by the team and codes were subsequently collapsed into overarching themes representing parent perceptions of the technology’s compatibility, utility, impact on child health and asthma management, and suggestions for improvement [21,22,24].

## Results

### Participant Characteristics

Characteristics of interview participants (parent-child dyads) are shown in Table 1. All but one parent identified as a mother. Most parents were college-educated, and there was an even mix of publicly and privately insured children.

**Table 1:** Characteristics of parent-child dyads (n=20).

Characteristics		Values
Child's age (years), mean (SE)		8.7 (0.6)
Child's sex (male), n (%)		14 (70)
**Child’s insurance, n (%)**		
	Public	10 (50)
	Private	10 (50)
**Parent's race, n (%)**		
	White	8 (40)
	African American or Black	7 (35)
	Asian	3 (15)
	Other	2 (10)
Hispanic ethnicity, n (%)		3 (15)
**Parent's education, n (%)**		
	Graduate/advanced degree	5 (25)
	College degree	9 (45)
	Some college/technical degree	3 (15)
	High school graduate/GED^a^	2 (10)
	Some high school	1 (5)
**Survey scores^b^, mean (SE)**		
	Asthma Control Test score (range 5-25)^c^	23.0 (0.7)
	Parental Asthma Management Self-Efficacy Scale score (range 1-5)	4.5 (0.1)
	Pediatric Asthma Caregiver’s Quality of Life Questionnaire score (range 1-7)	6.4 (0.3)
**Adherence level, n (%)**		
	Low (<30%)	6 (30)
	Medium (30%-70%)	8 (40)
	High (>70%)	6 (30)

^a^GED: General Education Diploma (ie, high school equivalency diploma).

^b^Scores are from surveys conducted at 12 months.

^c^Scores >19 indicate well-controlled asthma.

### Parental Experiences with EMM-Enhanced Intervention

Our qualitative analysis revealed the following 6 major themes regarding parents' experiences with the EMM-enhanced intervention: (1) compatibility with the family's lifestyle, (2) impact on asthma management, (3) impact on the child’s health, (4) emotional impact of the intervention, (5) child’s engagement in asthma management with the intervention, and (6) recommendations for future intervention design. Each theme is discussed below and exemplary quotes are provided in Table 2.

**Table 2:** Caregiver experiences and recommendations for an electronic medication monitoring intervention for pediatric asthma.

Themes	Exemplar quotes
Compatibility with lifestyle	“The fact that we’re all attached to our phones nowadays. Your face is constantly in your phone. You can’t miss it, it’s right there. Reminding you hey, it’s time to take your medicine or hey, he missed it this many times a week or you know hey, we noticed he had to take his albuterol more often.” [participant #91^a^, mother of an 8-year-old child]
Impact on asthma management	Prevention: “I’m so set now, I have that set schedule,…Because at first like I said we were like did I give it to him? I don’t know and it was like we know he needed it…life got in the way and we wouldn’t remember what we had done, so [now] it’s like it’s an automatic.” [participant #47, mother of a 6-year-old child] Acute management: “I think just patterns of increases use of rescue meds…then any time that we did have to you know intervene we could sort of see what was happening in the days leading up to that intervention and sort of figure out how to avoid those in the future.” [participant #37, mother of an 8-year-old child]
Impact on the child’s health	No change: “Right before we started using it he had already gone a good while without any asthma symptoms. So it’s hard to say whether this made that better or...if things would have continued on the same track.” [participant #48, mother of a 7-year-old child] Better health: “I think all of that really helped us stay on top of taking his medications so if he does catch a bug it’s not a long time that he’s sick.” [participant #91, mother of an 8-year-old child]
Emotional impact	Confidence: “I was a conscientious parent before the app, but the app certainly...helped me feel like I was more in control and build the confidence level of being knowledgeable about what’s going on with him and how to handle stuff.” [participant #15, mother of a 6-year-old child] Security (calls): “...makes me feel better that someone else is watching him as well and saying hey, we noticed this, you need to come in or...maybe you need to take him to the pediatrician or...hospital...I’m the primary caregiver and…administers the medication and watches over that, so knowing that someone else was there doing the same made me feel better.” [participant #91, mother of an 8-year-old child] Frustration: “...towards the end it...was not recording the Flovent. Like I would give it to her and it would say you have missed this dosage...and I’m like why does it keep saying that and I’ve given it to her and I had to keep resetting it…so that was sort of frustrating.” [participant #16, mother of an 11-year-old child]
Child engagement	“[He] really liked it. [He] was into getting into it and…make sure it showed that he did it and he’s like let’s look at the tips and he watched the different charts that we could see...he doesn’t get a lot of screen time, so anything that was on the phone (laughs) and it was about him, he was pretty excited about.” [participant #15, mother of a 6-year-old child]
Recommendations	“I think [the sensor and app] would work really well for parents that don’t have a lot of structure or capability to remember [when to give medications]. …I can’t tell you how many times I forgot or did without so people that don't, you know, have that knowledge or that share homes, you know they go from home to home.” [participant #79, mother of a 12-year-old child]

^a^Quotes are labeled with the dyad’s participant number from the original trial.

#### Compatibility With the Family's Lifestyle

Parents reported that using the technology was compatible with their daily schedules and daily cell phone use. Parents described the technology as “easy” because the app would show them whether their child had taken their medicine and reduced the need to ask their child repeatedly if they had taken their medicine. Parents appreciated that the technology could tell them if their child had used the rescue inhaler (ie, SABA) at school, as it can be difficult to find out from teachers and school staff if the medicine was taken. Parents reported that the app alerts were well-timed and served as a reminder to administer the medicine during hectic days. For example, some parents reported maintaining a more consistent medication schedule with the technology, as opposed to when they forgot to administer the medication or administered much later than prescribed on very hectic days.

On the other hand, parents also reported intervention barriers to compatibility, such as having multiple caregivers involved in the child’s asthma management, the involvement of grandparents unfamiliar with smartphone technology, and the intervention’s incompatibility when parents traveled out of town. Parents in families with multiple caregivers responsible for asthma management discussed how shared caregiving responsibilities made using the technology inconvenient:

Sometimes they might go to their grandparent’s house and we have to carry the sensor. Usually we have two different inhalers, one we kept at my in-laws' house and one over here, but if he’s using over there, he doesn’t have any sensor.

participant #118, father of an 11-year-old child

Further, these other caregivers were often grandparents, who parents noted were often unfamiliar with smartphones, as they might not own one themselves. Lastly, parents expressed some annoyance with not being able to sync the sensors when they traveled out of town without their child.

#### Impact on Asthma Management

Parents reported many aspects of the intervention that shaped their preventive and acute asthma management. For daily preventive management, parents reported improvement with app reminders, using the intervention to establish a routine or schedule that mostly endured after the study ended, using the pollen warnings to prepare for triggers, and having an increased awareness overall of their child’s asthma-related needs. Parents who had already established reliable asthma management routines before the intervention reported appreciating the technology but admitted that it did not change their behaviors.

For acute management, parents felt that one of the most useful features was the ability to track SABA use during asthma exacerbations. Parents reported that reviewing their child’s SABA use aided them in identifying triggers or patterns of asthma exacerbations. For example, a parent would not send their child outside to play on high trigger days because of pollen or weather changes. They also reported that the app replaced pen and paper or other previous methods in tracking SABA use. Parents described pulling up the app record for the doctor at clinic visits, enabling them to provide an accurate account to the doctor and preventing them from having to rely on their memory, which was less accurate.

#### Impact on the Child’s Health

Parents thought that the intervention was associated with improvements in their child’s health. Parents noted that they felt their child had more energy and fewer asthma attacks and that illness symptoms did not seem to last as long. Other parents, however, observed that their child had no change in their condition, reporting that the asthma was well-managed before the intervention or had improved with age. Only one parent suspected that their child’s asthma might have worsened over the course of the intervention; however, the parent emphasized that the technology and intervention made them more aware of the asthma and associated triggers and felt more capable of managing the asthma as a result.

#### Emotional Impact

A theme that emerged in the interviews was parents' emotional experience with the technology-enhanced intervention. Parents expressed a variety of emotions with using the intervention—confidence and a feeling of security but also occasional frustration. Many parents expressed feeling confident with the aid of the technology; they were better able to know what to do for an asthma exacerbation and would better remember to administer the medication before school and thus would not worry as much about their child’s asthma at school. Parents also felt more secure with a nurse monitoring their child’s medication use and were reassured when nurses or clinic staff would call to talk about their child’s asthma symptoms. On the other hand, parents also described frustration due to technical difficulties with syncing and tracking on the app. Also, one parent reported anxiety about being monitored: “Big brother is watching. We have to be good. We have to show them we can do this a little bit“ [participant #48, mother of a 7-year-old child].

#### Child's Engagement in Asthma Management

An unexpected theme that emerged was how the sensor and app promoted children's engagement in self-management. Parents reported that their child became engaged with taking care of their asthma because they were interested in the technology and app; for some parents, this led to a more active role for their child in their asthma management. Parents could assign their child a certain aspect of the asthma management responsibilities, such as pressing the sensor cap to give a dose of medication and watching its confirmation on the sensor light. Children’s engagement with the technology also included monitoring themselves on the app and playing with features on the app—doing quizzes, tracking puffs, and reading summaries and tips.

#### Recommendations

Parents had varying opinions on how to improve the intervention, the sensor technology, and its use. Parents identified that improving the sensor technology’s syncing capability was crucial; they reported that the “synchronizing issue” was difficult to resolve and were uncertain if the sensor had become “defective,” was “just a tech issue,” or was “disconnecting towards the end of the study…[because] it was the battery.”

To aid with follow-up phone calls from alerts, parents suggested incorporating texting in lieu of phone calls from health providers. Parents also expressed a desire for more app features that would engage children in their asthma management in an effort to reduce the need for parent prompting about medications in the future.

During the trial, families on Medicaid experienced a major change in managed care organization contracts, which led to insurance not covering certain inhaler medications that children had previously been prescribed. Thus, parents also asked that the sensor devices have greater compatibility with different inhaler medications.

When sharing who they believed the sensor system would work best for, parents recommended any parent or caregiver of a child with asthma. Others recommended the sensor system for those who might be newly diagnosed with asthma to help get them into a routine early on or for those with busy schedules who need reminders.

## Discussion

### Principal Findings

Our qualitative study, comprised of a purposive subsample of parents from a clinical trial, found that the EMM-based intervention was compatible with parents' daily lives, positively influenced their preventive and acute asthma management, and promoted children's engagement. Thus, overall, parents in our study found the intervention acceptable and generally favorable. However, parents also emphasized key improvements for the future design and development of this multicomponent, complex health intervention utilizing EMM [26,27].

Our qualitative work highlighted children's engagement as a key component of parents’ management of their child’s asthma through the EMM-based intervention. The app and sensors in particular seemed to provide a mechanism for parents to intentionally engage their child in the steps of asthma management. In pediatric health, the parent-child dyadic experience of the intervention may be a crucial factor driving perceptions of acceptability and potential adoption of new digital tools. Parents realized that children develop autonomy as they mature, but our findings also indicated that parents appreciated the early engagement of children to promote readiness for disease management in the future [28,29]. Future iterations of the mobile app program could include child-specific content through its features, such as tailoring of its tracking or quiz features to younger age groups, to encourage and sustain child engagement in asthma management. Parents further highlighted a desire for other digital features, such as videos or games, to engage their child in asthma education. While in-person asthma education is evidence-based and effective, digital delivery of asynchronous education could supplement and reinforce asthma education in the home setting for children and parents [30]. For example, digital feedback for asthma inhaler techniques is being explored as a replacement or supplement to qualitative feedback by in-person evaluation [31,32].

While the family's role in management of pediatric asthma has previously been well described, especially across urban minority families, parents described needing to change the way they coordinated asthma management with multiple caregivers when using the EMM-based intervention [33]. One prior study of inner-city families of children with asthma described that it is typical for up to four other caregivers to be involved in a child’s care and this sharing of asthma responsibilities can lead to unintended nonadherence to clinical recommendations [34]. In light of previous research and the present findings, we recommend that an adequate number of sensors be provided to each family. Additional education must also then be provided on how to download and manage apps on multiple phones for the same patient. This approach will account for multiple caregiver or blended family scenarios.

Next, many families in our study described dependence on family members, especially grandparents, as a source of caregiver support. Extended family caregivers are common in pediatric asthma, as suggested in a large patient study that found that 1 in 5 patients had an alternate caregiver living outside of the household who spent at least 6 hours per week with the child [35]. Parents, however, pointed to the generational gap in familiarity with digital technology. Overall, while seniors (ie, those older than 65 years) are adopting digital technologies at a much faster rate than in previous years, there are still noticeable differences in technology use according to age, income (ie, <$30,000 per household), and level of education (ie, high school education or lower) [36]. Supporting and educating families with extended generational caregiving of children is vital. For example, easy-to-access videos should be provided so that family members can educate each other on how to use the devices, rather than rely completely on clinical team support, and thus also reduce the burden on clinical staff [37].

Parents also expressed frustration or anxiety about how EMM interfaced with asthma management at home. The stress of caring for a child with a chronic disease is well described, and intervention design must be careful not to worsen the existing strain that families may already feel [38,39]. Issues around stress might be partly addressed by providing clearer communication about how to use the digital app and sensor and their limitations. For example, a few parents in our study expressed frustration with not being able to sync their app with the sensor when the devices were not in the same room. However, Bluetooth technology, the connection between the sensor and app, is a wireless, short-range communication, and thus parents should not have expected long-range functionality. Educating parents and health providers about the limits of the technology (ie, what to expect) through a built-in troubleshooting mechanism in the app may be useful to curb future frustrations.

Nevertheless, the stress that parents described may be primarily related to caring for a child with a chronic disease, and it is unclear whether a technology-enhanced intervention will alleviate that. At a minimum, more thorough assessments should be conducted to ensure that layering technology into parents’ asthma care management does not worsen their stress and anxiety, which in turn might worsen disease management [37]. Services for coordination and technology support are also necessary for clinical staff as they enroll families, explore their needs, and address how to use EMM appropriately for a range of family scenarios.

Parents also named specific improvements to the intervention design, including fixing syncing issues and using texts to mediate communication before phone calls. In addition to fixing the various syncing issues that parents noted, future intervention support is needed to help guide parents to handle errors they are experiencing with the devices. Parents turned to the research team for troubleshooting during the trial, but sustained implementation of the intervention will necessitate that the support roles of clinics and technology companies be clear to families or risk low adoption [37]. Parents also indicated that texting would be an acceptable intermediary step to speaking directly with the nurse or physician on the phone. Although texting should be acceptable to health providers for tracking ICS use because there is not an urgent medical need, further investigation as to the acceptability and feasibility of this approach among health providers regarding increased SABA use should be explored. In future iterations of the EMM-based intervention, texting could be considered as a first step for connecting with parents before initiating a direct conversation. An asynchronous approach for this component of the EMM-based intervention might alleviate the burden parents experience with trying to connect with the clinical team in a way that fits their busy schedules.

Given the varying experiences of and recommendations from parents, further research is needed to determine who this tool should be tailored for to support its optimal use. Factors that could be measured include technology literacy, the emotional burden of using the intervention over time, and potential changes in the home environment. Understanding and tracking these factors might aid the adaptation of EMM for clinical use while balancing patients’ preferences and needs.

### Limitations

Limitations to the study included a possible selection bias introduced by selecting parents who were willing to participate in the interviews. We tried to mitigate against selecting only parents with positive experiences by purposively sampling to achieve balanced representation of low, medium, and high adherence to daily therapy. However, we also wanted to include parents who had engaged in the intervention actively for 9 months, and this could have selected against parents who did not remain engaged with the intervention up to that time. Overall, the interviewed sample reflected the original trial sample in having children with controlled asthma, which limited our study from potentially capturing dyads who still experienced poorly controlled asthma. Given the limited sample size, we were not able to identify any distinct subthemes by different characteristics, such as insurance type or adherence level. Also, the amount of time between when parents exited the original trial and when they were interviewed for our study varied, and thus those who finished the trial longer ago may not have recalled their intervention experiences as accurately. The original trial also excluded non–English-speaking parents, which limits our understanding of parent experiences with EMM of non–English-speaking families. Further, 70% of the interview sample had a college degree or advanced degree, which reflects a highly educated interviewee participation.

### Conclusions

The parents’ perspective on the EMM-based intervention for asthma care was critical for understanding how a complex health intervention using technology could be improved or targeted in outpatient pediatric asthma care. While use of technology-enhanced tools is increasingly popular in health care delivery and consumer health care, our study highlighted that careful attention must be paid to the needs of parents of children with chronic diseases, such as asthma.

## Appendix

Multimedia Appendix 1 Interview guide questions.

## Abbreviations

EMM: electronic medication monitoring
ICS: inhaled corticosteroid
ICS-LABA: inhaled corticosteroid–long-acting beta-agonist
iTRACC: Improving Technology-Assisted Recording of Asthma Control in Children
SABA: short-acting beta-agonist
