# Supplementary material for: Parent Experiences With Electronic Medication Monitoring in Pediatric Asthma Management: Qualitative Study
Source: JMIR Pediatr Parent. 2021 Apr 23;4(2):e25811. doi: 10.2196/25811 (PMC8105758; doi:10.2196/25811)
Supplement: Multimedia Appendix 1 [file pediatrics_v4i2e25811_app1.docx]

Appendix Table 1. Interview Guide Questions

|  | Questions |
| --- | --- |
| Overall | What was your overall experience using the electronic sensor and app?  What did you like about the sensor and app? (Possible probe: Why?)  What did you not like about the sensor and app? (Possible probe: Why?) |
| Complexity | In what ways was the sensor and [app / hub] easy for you to use? (Possible probe: Why?)  What about the sensor and [app / hub] was difficult for you to use? |
| Compatibility | Can you tell me how using the sensor and app impacted your daily activities or schedule? |
| Relative Advantage | Do you see yourself using the sensor and app in the future to manage your child’s asthma?  Can you tell me how using the sensor and app changed the way you usually care for your child’s asthma? |
| Observability | Have you noticed any changes in your child’s asthma symptoms and health since you began using the sensor and app? |
| Recommendations | What were your expectations for using the sensor and app?  In what ways were your expectations [met / not met]?  Can you tell me what kind of information you got from the app?  What did you think about the information provided?  Can you tell me about any changes you have noticed in your relationship with your child’s asthma doctors since participating in the study?  Do you have any recommendations for how the sensor system and app/hub could be improved for future use? Why would you recommend these changes?  Would you recommend the sensor and app to a friend / another person [pick one] who has a child with asthma? (Probe for specific reasons why/why not).  Is there anything else you’d like to share about your experience using the sensor and app? |
